# Supplementary material for: Identification of Potential Antimalarial Drug Candidates Targeting Falcipain-2 Protein of Malaria Parasite—A Computational Strategy
Source: BioTech (Basel). 2022 Nov 30;11(4):54. doi: 10.3390/biotech11040054 (PMC9775493; doi:10.3390/biotech11040054)
Supplement: Supplementary file 1 [file biotech-11-00054-s001.zip › biotech-2049048-supplementary.pdf]

SUPPLEMENTARY: Identification of Potential Antimalarial Drug Candidates Targeting Falcipain-2 Protein of Malaria Parasite—A Computational Strategy

Shrikant Nema, Kanika Verma, Ashutosh Mani, Neha Shree Maurya, Archana Tiwari and Praveen Kumar Bharti

Table S1: Pharmacokinetic parameters for oral bioavailability evaluation of different lead molecules

S. No	Compounds Name	miLogP	MW	nON	nOHNH	nviolations
Ref.	Artemisinin	3.32	282.34	5	0	0
1	**Nicotine**	**1.09**	**162.24**	**2**	**0**	**0**
2	**Caffeine**	**0.06**	**194.19**	**6**	**0**	**0**
3	Reserpine	5.01	608.69	11	1	3
4	**Trospium**	**-0.61**	**392.52**	**4**	**1**	**0**
5	**Benzatropine**	**4.27**	**307.44**	**2**	**0**	**0**
6	**Methysergide**	**1.97**	**353.47**	**5**	**2**	**0**
7	**Cabergoline**	**3.44**	**451.62**	**7**	**2**	**0**
8	**Theophylline**	**-0.01**	**180.17**	**6**	**1**	**0**
9	**Morphine**	**1.1**	**285.34**	**4**	**2**	**0**
10	Vindesine	3.73	753.94	12	6	3
11	**Codeine**	**1.41**	**299.37**	**4**	**1**	**0**
12	Dihydroergotamine	2.09	583.69	10	3	1
13	**Hydromorphone**	**1.42**	**285.34**	**4**	**1**	**0**
14	**Ipratropium**	**-1.47**	**332.46**	**4**	**1**	**0**
15	**Methylergometrine**	**1.9**	**339.44**	**4**	**3**	**0**
16	Vinorelbine	5.92	778.95	12	2	3
17	Metocurine iodide	-1.41	652.83	8	0	1
18	**Miglustat**	**-0.72**	**219.28**	**5**	**4**	**0**
19	**Hyoscyamine**	**1.77**	**289.38**	**4**	**1**	**0**
20	**Quinine**	**3.06**	**324.42**	**4**	**1**	**0**
21	**Miglitol**	**-2.79**	**207.23**	**5**	**6**	**0**
22	**Oxycodone**	**0.79**	**315.37**	**5**	**1**	**0**
23	**Levallorphan**	**4.04**	**283.42**	**2**	**1**	**0**
24	**Dextromethorphan**	**3.94**	**271.4**	**2**	**0**	**0**
25	**Anisotropinemethylbromide**	**0.24**	**282.45**	**3**	**0**	**0**
26	Vincristine	4.95	824.97	14	3	2
27	Cisatracurium	-1.59	929.16	14	0	2

28	Vinblastine	5.56	810.99	13	3	3
29	**Atropine**	**1.77**	**289.38**	**4**	**1**	**0**
30	**Lisuride**	**3.16**	**338.45**	**5**	**2**	**0**
31	**Butorphanol**	**3.5**	**327.47**	**3**	**2**	**0**
32	**Dyphylline**	**-1.21**	**254.25**	**8**	**2**	**0**
33	**Pentazocine**	**4.59**	**285.43**	**2**	**1**	**0**
34	**Galantamine**	**1.54**	**287.36**	**4**	**1**	**0**
35	Ergotamine	2.08	581.67	10	3	1
36	**Nicergoline**	**3.85**	**484.39**	**6**	**0**	**0**
37	**Naltrexone**	**1.35**	**341.41**	**5**	**2**	**0**
38	**Apomorphine**	**2.89**	**267.33**	**3**	**2**	**0**
39	**Homatropinemethylbromide**	**-2.19**	**290.38**	**4**	**1**	**0**
40	Atracuriumbesylate	-1.59	929.16	14	0	2
41	**Scopolamine**	**1.05**	**303.36**	**5**	**1**	**0**
42	Irinotecan	4.1	586.69	10	1	1
43	**Pentoxifylline**	**0.65**	**278.31**	**7**	**0**	**0**
44	**Enprofylline**	**-0.14**	**194.19**	**6**	**2**	**0**
45	**Nalbuphine**	**1.71**	**357.45**	**5**	**3**	**0**
46	**Levorphanol**	**3.4**	**257.38**	**2**	**1**	**0**
47	**Cocaine**	**2.17**	**303.36**	**5**	**0**	**0**
48	**Quinidine**	**3.06**	**324.42**	**4**	**1**	**0**
49	**Buprenorphine**	**4.87**	**467.65**	**5**	**2**	**0**
50	**Hydrocodone**	**1.73**	**299.37**	**4**	**0**	**0**
51	**Physostigmine**	**1.94**	**275.35**	**5**	**1**	**0**
52	**Dimenhydrinate**	**3.5**	**255.36**	**2**	**0**	**0**
53	**Topotecan**	**2.03**	**421.45**	**8**	**2**	**0**
54	Ergoloidmesylate	2.83	611.74	10	3	1
55	**Pilocarpine**	**-0.24**	**208.26**	**4**	**0**	**0**
56	Deserpidine	4.98	578.66	10	1	1
57	**Papaverine**	**3.52**	**339.39**	**5**	**0**	**0**
58	Rescinnamine	5.43	634.73	11	1	3
59	**Naloxone**	**1.12**	**327.38**	**5**	**2**	**0**
60	**Pergolide**	**3.97**	**314.5**	**2**	**1**	**0**
61	**Oxymorphone**	**0.48**	**301.34**	**5**	**2**	**0**
62	Tubocurarine	1.99	609.74	8	2	1
63	Bromocriptine	3.6	654.61	10	3	1
64	**Aminophylline**	**-0.01**	**180.17**	**6**	**1**	**0**
65	**Ergometrine**	**1.36**	**325.41**	**5**	**3**	**0**
66	**Oxtriphylline**	**-3.55**	**179.16**	**6**	**0**	**0**
67	Metocurine	-1.41	652.83	8	0	1

68	**Yohimbine**	**3.22**	**354.45**	**5**	**2**	**0**
69	**Colchicine**	**1.1**	**399.44**	**7**	**1**	**0**
70	**Tiotropium**	**-1.94**	**392.52**	**5**	**1**	**0**
71	**Theobromine**	**-0.95**	**180.17**	**6**	**1**	**0**
72	**Ajmaline**	**2.56**	**326.44**	**4**	**2**	**0**
73	**5-Methoxy-N,N-diisopropyltryptamine**	**3.68**	**274.41**	**3**	**1**	**0**
74	**Dihydroetorphine**	**3.74**	**413.56**	**5**	**2**	**0**
75	**Diamorphine**	**1.61**	**369.42**	**6**	**0**	**0**
76	**Ethylmorphine**	**1.79**	**313.4**	**4**	**1**	**0**
77	**Cyprenorphine**	**3.52**	**423.55**	**5**	**2**	**0**
78	**Fenethylline**	**1.98**	**341.42**	**7**	**1**	**0**
79	**Etorphine**	**3.71**	**411.54**	**5**	**2**	**0**
80	**Benzoylecgonine**	**2.25**	**289.33**	**5**	**1**	**0**
81	**Ecgonine**	**-0.18**	**185.22**	**4**	**2**	**0**
82	**Desomorphine**	**2.76**	**271.36**	**3**	**1**	**0**
83	**Drotebanol**	**1.39**	**333.43**	**5**	**2**	**0**
84	**Diprenorphine**	**3.55**	**425.57**	**5**	**2**	**0**
85	**Dihydrocodeine**	**1.92**	**301.39**	**4**	**1**	**0**
86	**Dihydromorphine**	**1.61**	**287.36**	**4**	**2**	**0**
87	**Tropinone**	**0.29**	**139.2**	**2**	**0**	**0**
88	**8-azaxanthine**	**-1.23**	**153.1**	**7**	**3**	**0**
89	**7-Hydroxystaurosporine**	**3.42**	**482.54**	**8**	**3**	**0**
90	**Staurosporine**	**3.88**	**466.54**	**7**	**2**	**0**
91	**Xanthine**	**-1.09**	**152.11**	**6**	**3**	**0**
92	**K-252a**	**3.43**	**476.48**	**8**	**2**	**0**
93	**Duvoglustat**	**-2.4**	**163.17**	**5**	**5**	**0**
94	**Pseudotropine**	**0.48**	**141.21**	**2**	**1**	**0**
95	**Berberine**	**0.2**	**336.37**	**5**	**0**	**0**
96	**Arecoline**	**0.79**	**155.2**	**3**	**0**	**0**
97	**Methylecgonine**	**0.44**	**199.25**	**4**	**1**	**0**
98	**Camptothecin**	**2.03**	**348.36**	**6**	**1**	**0**
99	**Lysergic acid diethylamide**	**2.66**	**323.44**	**4**	**1**	**0**
100	Omacetaxinermepesuccinate	1.96	545.63	10	2	1
101	Voacamine	7.71	704.91	9	2	2
102	**Phenserine**	**3.64**	**337.42**	**5**	**1**	**0**
103	**Altropane**	**4.5**	**429.27**	**3**	**0**	**0**
104	**Migalastat**	**-2.4**	**163.17**	**5**	**5**	**0**
105	**Lobeline**	**3.73**	**337.46**	**3**	**1**	**0**
106	Vintafolide	-5.01	1917.07	47	25	3
107	**NS-2359**	**4.56**	**314.26**	**2**	**0**	**0**

108	**Cositecan**	**4.75**	**448.6**	**6**	**1**	**0**
109	**Rivanicline**	**1.3**	**162.24**	**2**	**1**	**0**
110	**Rubitecan**	**2.24**	**393.36**	**9**	**1**	**0**
111	**Nalmefene**	**2.76**	**339.44**	**4**	**2**	**0**
112	**Naxifylline**	**2.65**	**344.42**	**7**	**1**	**0**
113	**Propentofylline**	**1.53**	**306.37**	**7**	**0**	**0**
114	Midostaurin	4.69	570.65	8	1	1
115	**Gimatecan**	**3.24**	**447.49**	**8**	**1**	**0**
116	**Sparteine**	**2.9**	**234.39**	**2**	**0**	**0**
117	**Drotaverine**	**4.54**	**397.51**	**5**	**1**	**0**
118	**Capsaicin**	**3.1**	**305.42**	**4**	**2**	**0**
119	**Methylnaltrexone**	**-2.63**	**356.44**	**5**	**2**	**0**
120	**3-isobutyl-1-methyl-7H-xanthine**	**1.12**	**222.25**	**6**	**1**	**0**
121	**Ioflupane I-123**	**4.8**	**431.29**	**3**	**0**	**0**
122	**Uric acid**	**-1.44**	**168.11**	**7**	**4**	**0**
123	**Aclidinium**	**0.59**	**484.66**	**5**	**1**	**0**
124	**Fentonium**	**0.73**	**484.62**	**5**	**1**	**0**
125	Naloxegol	0.5	651.79	12	2	2
126	**Xanthinol**	**-1.18**	**311.34**	**9**	**2**	**0**
127	**Zucapsaicin**	**3.1**	**305.42**	**4**	**2**	**0**
128	**Pholcodine**	**1.29**	**398.5**	**6**	**1**	**0**
129	**Cimetropium**	**-2.06**	**358.46**	**5**	**1**	**0**
130	**Doxofylline**	**-0.13**	**266.26**	**8**	**0**	**0**
131	**Butylscopolamine**	**-1.49**	**360.47**	**5**	**1**	**0**
132	**Homatropine**	**1.78**	**275.35**	**4**	**1**	**0**
133	Dihydroergocornine	2.15	563.7	10	3	1
134	Epicriptine	2.65	577.73	10	3	1
135	**Methscopolamine**	**-2.93**	**318.39**	**5**	**1**	**0**
136	**Nonivamide**	**4.12**	**293.41**	**4**	**2**	**0**
137	**Nalorphine**	**1.75**	**311.38**	**4**	**2**	**0**
138	**Metoserpate**	**3.57**	**428.53**	**7**	**1**	**0**
139	**Bicuculline**	**2.88**	**367.36**	**7**	**0**	**0**
140	Thiocolchicoside	-0.16	563.62	11	5	2
141	Vinflunine	6.24	816.94	12	2	3
142	**Psilocybin**	**1.02**	**284.25**	**6**	**3**	**0**
143	**Anisodamine**	**0.85**	**305.37**	**5**	**2**	**0**
144	**Axelopran**	**2.35**	**457.62**	**7**	**4**	**0**
145	**Oxitropium**	**-2.56**	**332.42**	**5**	**1**	**0**
146	**Tetrahydropalmatine**	**2.75**	**355.43**	**5**	**0**	**0**
147	**Namitecan**	**0.86**	**434.45**	**9**	**3**	**0**

148	**Vinpocetine**	**4.12**	**350.46**	**4**	**0**	**0**
149	**Exatecan**	**0.75**	**435.45**	**7**	**3**	**0**
150	Lurtotecan	1.44	518.57	10	1	1
151	**Moxaverine**	**4.88**	**307.39**	**3**	**0**	**0**
152	**AR-67**	**4.556**	**478.62**	**7**	**2**	**0**
153	**10-hydroxycamptothecin**	**1.52**	**364.36**	**7**	**2**	**0**
154	**Lisofylline**	**0.84**	**280.33**	**7**	**1**	**0**
155	Zalypsis	4.87	709.72	11	3	2
156	**Belotecan**	**2.9**	**433.51**	**7**	**2**	**0**
157	**9-aminocamptothecin**	**1.77**	**363.37**	**7**	**3**	**0**
158	**Samidorphan**	**1.31**	**370.45**	**6**	**4**	**0**
159	**Tonapofylline**	**3.1**	**416.52**	**8**	**2**	**0**
160	Patidegib	5.73	504.78	5	2	2
161	**Rolofylline**	**3.95**	**356.47**	**4**	**1**	**0**
162	Solamargine	2.41	868.07	16	9	3
163	Pegamotecan	3.38	980.98	20	2	2
164	**Reproterol**	**-0.45**	**389.41**	**10**	**4**	**0**
165	Sacituzumabgovitecan	-3.19	1601.8	36	10	3
166	**Cafedrine**	**0.99**	**357.41**	**8**	**2**	**0**
167	**Theodrenaline**	**-0.66**	**375.38**	**10**	**4**	**0**
168	**8-cyclopentyl-1,3-dipropylxanthine**	**3.24**	**304.39**	**6**	**1**	**0**
169	CT-2584	7.9	533.8	8	2	2
170	**Ambroxolacefyllinate**	**2.74**	**378.11**	**3**	**4**	**0**
171	**Bamifylline**	**1.9**	**385.47**	**8**	**1**	**0**
172	Atracurium	-1.59	929.16	14	0	2
173	**Demecolcine**	**1.74**	**371.43**	**6**	**1**	**0**
174	Dihydroergocristine	2.83	611.74	10	3	1
175	**Vincamine**	**3.18**	**354.45**	**5**	**1**	**0**
176	Dihydroergocryptine	2.68	577.73	10	3	1
177	**Emetine**	**3.64**	**480.65**	**6**	**1**	**0**
178	**Terguride**	**3.17**	**340.47**	**5**	**2**	**0**
179	**Proxyphylline**	**-0.21**	**238.25**	**7**	**1**	**0**
180	**Nicomorphine**	**2.5**	**495.54**	**8**	**0**	**0**
181	**Etybenzatropine**	**4.65**	**321.46**	**2**	**0**	**0**
182	**Nalfurafine**	**2.63**	**476.57**	**7**	**2**	**0**
183	**Metergoline**	**3.87**	**403.53**	**5**	**1**	**0**
184	**Prajmaline**	**-0.54**	**369.53**	**4**	**2**	**0**
185	**Acefylline**	**-0.69**	**238.2**	**8**	**1**	**0**
186	Bietaserpine	5.86	707.87	12	0	3
187	Etamiphylline	0.84	279.34	7	0	0

188	**Phenazocine**	**4.72**	**321.46**	**2**	**1**	**0**
189	**Pentifylline**	**2.51**	**264.33**	**6**	**0**	**0**
190	Alcuronium	-2.45	666.91	6	2	1
191	**Lorajmine**	**3.66**	**402.92**	**5**	**1**	**0**
192	**Tretoquinol**	**2.12**	**345.339**	**6**	**3**	**0**
193	**Hydroquinine**	**3.29**	**326.44**	**4**	**1**	**0**
194	**Vinburnine**	**3.78**	**294.4**	**3**	**0**	**0**
195	**Dimemorfan**	**4.33**	**255.41**	**1**	**0**	**0**
196	**Bufylline**	**-0.01**	**180.17**	**6**	**1**	**0**
197	**Methylatropine**	**-2.21**	**304.41**	**4**	**1**	**0**
198	**Dehydroemetine**	**3.3**	**478.63**	**6**	**1**	**0**
199	**Harmaline**	**2.68**	**214.27**	**3**	**1**	**0**
200	**Bromotheophylline**	**1.12**	**259.06**	**6**	**1**	**0**
201	**Furafylline**	**0.94**	**260.25**	**7**	**1**	**0**
202	Tetrandrine	6.55	622.76	8	0	2
203	**8-chlorotheophylline**	**0.99**	**214.61**	**6**	**1**	**0**
204	**Dextrorphan**	**3.4**	**257.38**	**2**	**1**	**0**
205	**7-ethyl-10-hydroxycamptothecin**	**2.67**	**392.41**	**7**	**2**	**0**
206	**Rauwolfiaserpentina root**	**3.41**	**352.43**	**5**	**1**	**0**
207	Almitrine	6.14	477.56	7	2	1
208	**Morphine glucuronide**	**-0.73**	**461.47**	**10**	**5**	**0**
209	Firtecanpegol	7.22	2300.32	49	8	4
210	**18-methoxycoronaridine**	**3.55**	**368.48**	**5**	**1**	**0**
211	**Hydroquinidine**	**3.29**	**326.44**	**4**	**1**	**0**
212	**Posiphen**	**3.64**	**337.42**	**5**	**1**	**0**
213	**Dihydrocapsiate**	**4.74**	**308.42**	**4**	**1**	**0**
214	Naldemedine	3.65	570.65	10	4	1
215	**Deptropine**	**4.39**	**333.48**	**2**	**0**	**0**
216	**Tenidap**	**3.34**	**320.76**	**5**	**3**	**0**
217	Loxicodegol	0.36	595.73	11	1	2
218	**Lucerastat**	**-0.72**	**219.28**	**5**	**4**	**0**
219	**PCS-499**	**0.84**	**280.337**	**7**	**1**	**0**
220	**Colchiceine**	**0.83**	**385.42**	**7**	**2**	**0**
221	**Variolin B**	**-0.15**	**293.29**	**8**	**5**	**0**
222	Deacetoxyvinzolidine	6.21	826.43	12	2	3
223	**Harmine**	**2.63**	**212.25**	**3**	**1**	**0**
224	Raubasine	2.83	611.74	10	3	1
225	**Strychnine**	**1.84**	**334.42**	**4**	**0**	**0**
226	**Butropium**	**0.88**	**452.62**	**5**	**1**	**0**
227	**Diflomotecan**	**1.25**	**398.37**	**6**	**1**	**0**

228	**Nazartinib**	**3.28**	**495.03**	**8**	**1**	**0**
229	Dihydroergotoxine	2.09	583.69	10	3	1
230	3,5'-dihydroxythalifaboramine	5.9	684.79	11	3	3
231	3-hydroxy-6'-desmethyl-9-O-methylthalifaboramine	5.96	668.79	10	2	2
232	3-hydroxythalifaboramine	5.99	668.79	10	2	2
233	5'-hydroxythalifaboramine	5.99	668.79	10	2	2
234	6'-desmethylthalifaboramine	5.78	638.76	9	2	2
235	Alpha-tomatine	-0.92	1034.2	22	13	3
236	Berbamine	6.24	608.74	8	1	2
237	**Berberine**	**0.2**	**336.37**	**5**	**0**	**0**
238	Betanin	-6.03	550.47	15	8	3
239	**Brucine**	**1.46**	**394.47**	**6**	**0**	**0**
240	**Brucine N-oxide**	**1.42**	**410.47**	**7**	**0**	**0**
241	**Camptothecin**	**2.03**	**348.36**	**6**	**1**	**0**
242	**Chelerythrine**	**0.75**	**348.38**	**5**	**0**	**0**
243	**Cis-Zeatin**	**1.04**	**219.25**	**6**	**3**	**0**
244	**Cryptolepine**	**3.86**	**232.29**	**2**	**0**	**0**
245	Dehydrotomatine	-1.1	1032.18	22	13	3
246	Emarginatine F	3.64	918.9	20	2	2
247	Emarginatine G	3.3	924.91	21	1	2
248	Fangchinoline	6.27	608.74	8	1	2
249	Harmaline	6.54	409.19	3	0	1
250	**Harmalol**	**3.02**	**323.35**	**6**	**2**	**0**
251	**Harmane**	**2.59**	**182.23**	**2**	**1**	**0**
252	**Harmine**	**2.63**	**212.25**	**3**	**1**	**0**
253	**Harmol**	**1.89**	**198.22**	**3**	**2**	**0**
254	Homoharringtonine	1.96	545.63	10	2	1
255	**Hydrastine**	**2.83**	**383.4**	**7**	**0**	**0**
256	**Isostrychnine**	**1.46**	**334.42**	**4**	**1**	**0**
257	**Lycoricidinol**	**-1.12**	**307.26**	**8**	**5**	**0**
258	**Lycorine**	**0.54**	**287.31**	**5**	**2**	**0**
259	Mahanimbicine	7.15	331.46	2	1	1
260	Mahanimbine	7.1	331.46	2	1	1
261	Mahanine	6.15	333.43	3	2	1
262	**Marcanines A**	**1.92**	**239.23**	**4**	**1**	**0**
263	**Matrine**	**1.98**	**248.37**	**3**	**0**	**0**
264	**N-(4-hydroxyundecanoyl)anabasine**	**4.27**	**346.51**	**4**	**1**	**0**
265	**Neocryptolepine**	**3.86**	**232.29**	**2**	**0**	**0**
266	**N-n-octanoylnornicotine**	**3.87**	**274.41**	**3**	**0**	**0**

267	**Noscapine**	**2.81**	**413.43**	**8**	**0**	**0**
268	**Papaverine**	**3.52**	**339.39**	**5**	**0**	**0**
269	**Physostigmine**	**1.94**	**275.35**	**5**	**1**	**0**
270	**Reserpic acid**	**2.33**	**400.48**	**7**	**3**	**0**
271	**Rhyncophylline**	**2.83**	**384.48**	**6**	**1**	**0**
272	**Rohitukine**	**1.13**	**305.33**	**6**	**3**	**0**
273	**Sanguinarine**	**0.8**	**332.33**	**5**	**0**	**0**
274	Solamargine	2.41	868.07	16	9	3
275	**Strychnine**	**1.84**	**334.42**	**4**	**0**	**0**
276	Tetrandrine	6.55	622.76	8	0	2
277	Tomatidenol	5.73	413.65	3	2	1
278	Tomatidine	5.92	415.66	3	2	1
279	Vinblastine	5.56	810.99	13	3	3
280	**Vincamine**	**3.18**	**354.45**	**5**	**1**	**0**
281	Vincristine	4.95	824.97	14	3	2
282	**(-)-anonaine**	**3.25**	**265.31**	**3**	**1**	**0**
283	**(+)-tylophorinidine**	**2.75**	**365.43**	**5**	**2**	**0**
284	**(R)-antofine**	**4.05**	**363.46**	**4**	**0**	**0**
285	**(R)-cryptopleurine**	**4.55**	**377.48**	**4**	**0**	**0**
286	**(R)-tylophorine**	**3.64**	**393.48**	**5**	**0**	**0**
287	**(S)-tyloindicine I**	**2.93**	**409.48**	**6**	**1**	**0**
288	**11-beta-hydroxycephalotaxine beta-N-oxide**	**-0.04**	**347.37**	**7**	**2**	**0**
289	5alpha-O-(3'-dimethylamino-3'-phenylpropionyl) taxinine M	4.78	861.94	16	1	2
290	**9-Aminocamptothecin**	**1.77**	**363.37**	**7**	**3**	**0**
291	**9-methoxycanthin-6-one**	**2.72**	**250.26**	**4**	**0**	**0**
292	**9-methoxycanthin-6-one 3N-oxide**	**1.31**	**266.26**	**5**	**0**	**0**
293	**Antofine**	**4.05**	**363.46**	**4**	**0**	**0**
294	**beta-carboline-1-propionic acid**	**2.31**	**240.26**	**4**	**2**	**0**
295	**canthin-6-one**	**2.69**	**220.23**	**3**	**0**	**0**
296	**canthin-6-one 9-O-beta-glucopyranoside**	**0.4**	**398.37**	**9**	**4**	**0**
297	**Capsaicin**	**3.1**	**305.42**	**4**	**2**	**0**
298	**Cephalotaxine alpha-N-oxide**	**0.95**	**331.37**	**6**	**1**	**0**
299	**Cephalotaxine beta-N-oxide**	**0.95**	**331.37**	**6**	**1**	**0**
300	Cepharanthine	6.59	606.72	8	0	2
301	**Corydaline**	**3.08**	**369.46**	**5**	**0**	**0**
302	Cycleanine	6.75	622.76	8	0	2
303	**Ellipticine**	**4.28**	**246.31**	**2**	**1**	**0**
304	**Evodiamine**	**2.82**	**303.37**	**4**	**1**	**0**
305	**Holacurtine**	**4.04**	**491.71**	**6**	**2**	**0**

306	**Holacurtinol**	**2.04**	**491.67**	**7**	**4**	**0**
307	**Holamine**	**3.5**	**315.5**	**2**	**2**	**0**
308	**Isocephalotaxine**	**1**	**315.37**	**5**	**1**	**0**
309	Isotetrandrine	6.55	622.76	8	0	2
310	**liriodenine**	**3.3**	**275.26**	**4**	**0**	**0**
311	**Psychotrine**	**4.71**	**478.63**	**6**	**0**	**0**
312	**Secoantofine**	**3.76**	**365.47**	**4**	**0**	**0**
313	**Tubulosine**	**4.86**	**475.63**	**6**	**3**	**0**
314	**Tylocrebrine**	**3.84**	**393.48**	**5**	**0**	**0**
315	**tyloindicine F**	**3.65**	**381.47**	**5**	**1**	**0**
316	**tyloindicine G**	**2.65**	**409.48**	**6**	**1**	**0**
317	**tylophoridicine C**	**2.71**	**381.43**	**6**	**2**	**0**
318	**tylophoridicine F**	**3.02**	**395.45**	**6**	**1**	**0**
319	Vincristine sulfate	4.95	824.97	14	3	2
320	**(S)-tylophorine**	**3.64**	**393.48**	**5**	**0**	**0**
321	**6-O-desmethylantofine**	**3.51**	**349.43**	**4**	**1**	**0**
322	**13(R)-antofine-N-oxide**	**4.01**	**379.46**	**5**	**0**	**0**
323	**13(R)-14(R)-hydroxyantofine-N-oxide**	**3.02**	**395.45**	**6**	**1**	**0**
324	**7-methoxy-beta-carboline-1-propionic acid**	**2.34**	**270.29**	**5**	**2**	**0**
325	**4-methoxycarbonyl-5,10-benzogquinolinequinone**	**2.08**	**267.24**	**5**	**0**	**0**
326	**6-O-desmethylsecoantofine**	**3.22**	**351.45**	**4**	**1**	**0**
327	**Saprosmine A**	**2.37**	**285.3**	**5**	**1**	**0**
328	**Saprosmine B**	**2.75**	**299.33**	**5**	**1**	**0**
329	2-alpha-acetoxy-2'beta-deacetylaustrospicatine	5.89	783.91	14	1	3
330	**9-hydroxycanthin-6-one**	**1.99**	**236.23**	**4**	**1**	**0**
331	**15-alpha-Hydroxyholamine**	**2.58**	**331.5**	**3**	**3**	**0**
332	**17-epi-Holacurtine**	**4.04**	**491.71**	**6**	**2**	**0**
333	**3-alpha-Amino-14-beta-hydroxypregnan-20-one**	**2.74**	**333.52**	**3**	**3**	**0**
334	**17-epi-N-Demethylholacurtine**	**3.13**	**477.69**	**6**	**3**	**0**
335	**N-Demethylholacurtine**	**3.13**	**477.69**	**6**	**3**	**0**
336	**(S)-isotylocrebrine**	**3.84**	**393.48**	**5**	**0**	**0**
337	**Isotylocrebrine**	**3.84**	**393.48**	**5**	**0**	**0**
338	**7-desmethyltylophorine**	**3.33**	**379.46**	**5**	**1**	**0**
339	1',2',3',4'-tetradehydrotubulosine	5.26	471.6	6	2	1
340	**Reticuline**	**2.38**	**329.4**	**5**	**2**	**0**

Note: Bold entries were taken for further analysis

Table S2: Drug-likeness and Toxicity risk assessment of Artemisinin and alkaloids

S. No.	Compounds Name	Druglikeness	Mutagenic	Tumorigenic	Reproductive Effective	Irritant
Ref	Artemisinin	-1.97	None	None	High	High
1	Uric acid	2.923	high	none	high	none
2	**Xanthine**	**3.2262**	**none**	**none**	**none**	**none**
3	**8-cyclopentyl-1,3-dipropylxanthine**	**2.6064**	**none**	**none**	**none**	**none**
4	Enprofylline	5.7339	high	none	none	none
5	Theophylline	2.5134	high	high	high	none
6	Arecoline	3.0974	high	high	none	none
7	Berberine	-2.2467	none	none	none	none
8	Caffeine	2.5858	high	high	high	none
9	Nonivamide	-19.466	none	none	none	none
10	**Dyphylline**	**4.4457**	**none**	**none**	**none**	**none**
11	**Furafylline**	**4.374**	**none**	**none**	**none**	**none**
12	Harmaline	2.0945	none	none	high	none
13	3-isobutyl-1-methyl-7H-xanthine	-1.4031	high	none	none	none
14	Papaverine	-1.7454	none	none	none	none
15	Pentoxifylline	-1.5832	high	none	high	high
16	Propentofylline	-1.6163	none	none	none	high
17	Proxyphylline	2.7251	low	high	none	none
18	Tetrahydropalmatine	2.9203	none	none	low	none
19	Theobromine	3.421	high	high	high	none
20	Lysergic acid diethylamide	7.8391	none	none	high	none

21	**Pilocarpine**	**4.1824**	**none**	**none**	**none**	**none**
22	**Physostigmine**	**1.9297**	**none**	**none**	**none**	**none**
23	**Apomorphine**	**4.7822**	**none**	**none**	**none**	**none**
24	Colchicine	1.024	none	none	high	none
25	Methylergometrine	8.0208	none	none	high	high
26	**Pseudotropine**	**1.8332**	**none**	**none**	**none**	**none**
27	**Yohimbine**	**1.5035**	**none**	**none**	**none**	**none**
28	Aminophylline	2.5134	high	high	high	none
29	**Galantamine**	**5.3857**	**none**	**none**	**none**	**none**
30	Methysergide	8.0208	high	none	high	high
31	Xanthinol	**6.0082**	**none**	**none**	**none**	**none**
32	Emetine	3.8313	none	high	none	high
33	Psilocybin	-14.96	high	none	none	none
34	Dimenhydrinate	4.6945	high	none	high	none
35	**8-chlorotheophylline**	**2.5231**	**none**	**none**	**none**	**none**
36	**Bromotheophylline**	**0.68159**	**none**	**none**	**none**	**none**
37	Phenazocine	3.329	none	none	high	none
38	**Vincamine**	**1.8025**	**none**	**none**	**none**	**none**
39	**Bamifylline**	**5.8382**	**none**	**none**	**none**	**none**
40	Fenethylline	7.1692	none	none	high	none
41	Dehydroemetine	3.8992	none	high	none	high
42	Bufylline	2.5134	high	high	high	none
43	**Camptothecin**	**5.3292**	**none**	**none**	**none**	**none**
44	Reproterol	5.7176	none	none	high	none
45	Etamiphylline	6.8977	none	none	none	high

46	Metergoline	-7.3817	none	none	high	none
47	Lisuride	7.3646	none	none	high	none
48	**Duvoglustat**	**3.055**	**none**	**none**	**none**	**none**
49	Nicergoline	2.5093	none	none	high	none
50	**Staurosporine**	**4.7259**	**none**	**none**	**none**	**none**
51	Pergolide	4.1164	none	none	high	none
52	Doxofylline	-2.5282	none	none	none	none
53	**Miglustat**	**2.5386**	**none**	**none**	**none**	**none**
54	Cabergoline	6.1842	none	none	high	none
55	**Topotecan**	**6.6593**	**none**	**none**	**none**	**none**
56	Tenidap	5.0482	none	high	none	high
57	**Rolofylline**	**5.4469**	**none**	**none**	**none**	**none**
58	**Tretoquinol**	**1.2531**	**none**	**none**	**none**	**none**
59	Metoserpate	2.1823	none	none	high	none
60	Acefylline	-2.1888	none	none	none	none
61	Pentifylline	-5.5075	none	none	none	none
62	Moxaverine	-2.0047	none	none	none	none
63	**Methscopolamine**	**1.9941**	**none**	**none**	**none**	**none**
64	**Vinburnine**	**3.4076**	**none**	**none**	**none**	**none**
65	Cafedrine	6.7504	none	none	low	none
66	**Theodrenaline**	**5.6468**	**none**	**none**	**none**	**none**
67	**7-Hydroxystaurosporine**	**5.0622**	**none**	**none**	**none**	**none**
68	9-aminocamptothecin	5.3457	high	low	none	none
69	**Tropinone**	**1.4196**	**none**	**none**	**none**	**none**
70	**Nicotine**	**4.4595**	**none**	**none**	**none**	**none**

71	**Ecgonine**	**1.8158**	**none**	**none**	**none**	**none**
72	Hydroquinidine	1.038	none	none	none	high
73	10-hydroxycamptothecin	**5.3292**	**none**	**none**	**none**	**none**
74	Lobeline	-0.2215	none	none	none	none
75	**7-ethyl-10-hydroxycamptothecin**	**5.3292**	**none**	**none**	**none**	**none**
76	Dihydroetorphine	3.265	none	none	none	high
77	Hydroquinine	1.038	none	none	none	high
78	Cositecan	-68.377	none	none	none	high
79	**Exatecan**	**3.0211**	**none**	**none**	**none**	**none**
80	**5-Methoxy-N,N-diisopropyltryptamine**	**3.0812**	**none**	**none**	**none**	**none**
81	**Hyoscyamine**	**3.0135**	**none**	**none**	**none**	**none**
82	Butylscopolamine	-0.38167	none	none	none	none
83	**Atropine**	**3.0135**	**none**	**none**	**none**	**none**
84	**Migalastat**	**3.055**	**none**	**none**	**none**	**none**
85	Ambroxolacefyllinate	-1.2867	none	none	none	none
86	Phenserine	-3.6765	none	none	none	none
87	**Deptropine**	**2.1325**	**none**	**none**	**none**	**none**
88	**Tonapofylline**	**4.1019**	**none**	**none**	**none**	**none**
89	**Diflomotecan**	**2.7696**	**none**	**none**	**none**	**none**
90	Demecolcine	1.837	none	none	high	none
91	Colchiceine	1.1911	high	none	high	none
92	Methylecgonine	-0.18672	none	none	none	none
93	**Noscapine**	**4.3358**	**none**	**none**	**none**	**none**
94	**Strychnine**	**4.5843**	**none**	**none**	**none**	**none**
95	**Quinidine**	**0.87864**	**none**	**none**	**none**	**none**

96	Pentazocine	3.3217	none	none	high	none
97	**Miglitol**	**4.2706**	**none**	**none**	**none**	**none**
98	**Rauwolfiaserpentina root**	**2.6043**	**none**	**none**	**none**	**none**
99	**Diprenorphine**	**2.5267**	**none**	**none**	**none**	**none**
100	Ergometrine	6.2554	none	none	high	none
101	Terguride	7.3416	none	none	high	none
102	Vinpocetine	-0.26904	none	none	none	none
103	Cocaine	-0.52972	none	none	high	high
104	**Benzoylecgonine**	**1.4683**	**none**	**none**	**none**	**none**
105	**Rubitecan**	**0.38166**	**none**	**none**	**none**	**none**
106	Lisofylline	-3.3518	none	none	none	none
107	**Lucerastat**	**2.5386**	**none**	**none**	**none**	**none**
108	**Sparteine**	**1.4482**	**none**	**none**	**none**	**none**
109	**Buprenorphine**	**2.5141**	**none**	**none**	**none**	**none**
110	Etorphine	4.4752	none	none	none	high
111	Butropium	-3.2621	none	none	none	high
112	Methylatropine	3.4926	none	none	none	none
113	**Oxtriphylline**	**1.7125**	**none**	**none**	**none**	**none**
114	**Anisotropinemethylbromide**	**1.7176**	**none**	**none**	**none**	**none**
115	**Ipratropium**	**1.8233**	**none**	**none**	**none**	**none**
116	**Benzatropine**	**2.1325**	**none**	**none**	**none**	**none**
117	Zucapsaicin	-10.379	none	none	none	none
118	Capsaicin	-10.379	none	none	none	none
119	Drotaverine	-0.30443	none	none	high	none
120	**Scopolamine**	**2.875**	**none**	**none**	**none**	**none**

121	**Quinine**	**0.87864**	**none**	**none**	**none**	**none**
122	**K-252a**	**1.9148**	**none**	**none**	**none**	**none**
123	Dimemorfan	-0.22762	none	none	none	none
124	Ioflupane I-123	-1.1542	none	none	none	none
125	Harmine	-0.92948	low	none	none	none
126	Homatropine	1.1497	none	none	none	high
127	**Codeine**	**5.1305**	**none**	**none**	**none**	**none**
128	**Dihydrocodeine**	**4.2758**	**none**	**none**	**none**	**none**
129	**Hydrocodone**	**4.2643**	**none**	**none**	**none**	**none**
130	**Hydromorphone**	**4.2337**	**none**	**none**	**none**	**none**
131	**Nalmefene**	**1.35**	**none**	**none**	**none**	**none**
132	**Nalorphine**	**0.91284**	**none**	**none**	**none**	**none**
133	**Naloxone**	**0.78037**	**none**	**none**	**none**	**none**
134	**Oxycodone**	**4.9034**	**none**	**none**	**none**	**none**
135	**Oxymorphone**	**4.8562**	**none**	**none**	**none**	**none**
136	Trospium	-0.46271	none	none	none	none
137	**Morphine**	**5.0881**	**none**	**none**	**none**	**none**
138	Rivanicline	-0.28957	none	high	none	none
139	**Nalbuphine**	**3.6567**	**none**	**none**	**none**	**none**
140	**Bicuculine**	**0.12**	**None**	**None**	**None**	**None**
141	**Pholcodine**	**5.8282**	**none**	**none**	**none**	**none**
142	**Ethylmorphine**	**3.6915**	**none**	**none**	**none**	**none**
143	Levorphanol	-0.25467	none	none	low	none
144	Levallorphan	-4.6071	none	none	none	none
145	**Dihydromorphine**	**4.2386**	**none**	**none**	**none**	**none**

146	**Naltrexone**	**4.6068**	**none**	**none**	**none**	**none**
147	**Morphine glucuronide**	**3.962**	**none**	**none**	**none**	**none**
148	Dextromethorphan	-0.33388	none	none	none	none
149	Dextrorphan	-0.25467	none	none	low	none
150	Butorphanol	-0.62733	none	none	none	none
151	Methylnaltrexone	0.63257	none	none	none	none
152	**Desomorphine**	**2.0446**	**none**	**none**	**none**	**none**
153	**Nicomorphine**	**4.7938**	**none**	**none**	**none**	**none**
154	**Diamorphine**	**4.8851**	**none**	**none**	**none**	**none**
155	**Drotebanol**	**4.6517**	**none**	**none**	**none**	**none**
156	**Tiotropium**	**4.8619**	**none**	**none**	**none**	**none**
157	**Ajmaline**	**3.4513**	**none**	**none**	**none**	**none**
158	Altropane	-1.3287	none	none	none	none
159	**Nalfurafine**	**4.4373**	**none**	**none**	**none**	**none**
160	**Belotecan**	**7.6567**	**none**	**none**	**none**	**none**
161	AR-67	-52.664	none	none	none	high
162	Oxitropium	**1.9563**	**none**	**none**	**none**	**none**
163	Anisodamine	3.4327	none	none	none	none
164	Gimatecan	-2.2795	none	none	none	none
165	**Variolin B**	**3.1147**	**none**	**none**	**none**	**none**
166	**Naxifylline**	**5.639**	**none**	**none**	**none**	**none**
167	Dihydrocapsiate	-15.31	none	none	none	none
168	**Fentonium**	**1.9364**	**none**	**none**	**none**	**none**
169	Homatropinemethylbromide	1.3761	none	none	none	high
170	Namitecan	5.1799	none	none	none	none

171	Posiphen	-3.6765	none	none	none	none
172	**Aclidinium**	**3.1287**	**none**	**none**	**none**	**none**
173	Samidorphan	4.4951	none	none	none	none
174	**18-methoxycoronaridine**	**1.6643**	**none**	**none**	**none**	**none**
175	**Cimetropium**	**2.22**	**none**	**none**	**none**	**none**
176	**Cyprenorphine**	**3.7547**	**none**	**none**	**none**	**none**
177	**Etybenzatropine**	**1.0379**	**none**	**none**	**none**	**none**
178	**PCS-499**	**3.9575**	**none**	**none**	**none**	**none**
179	**Axelopran**	**0.25891**	**none**	**none**	**none**	**none**
180	**NS-2359**	**0.98602**	**none**	**none**	**none**	**none**
181	Nazartinib	2.8132	none	high	high	none
182	Lorajmine	2.1446	high	high	high	high
183	**Prajmaline**	**2.3465**	**none**	**none**	**none**	**none**
184	**8-azaxanthine**	**4.4521**	**none**	**none**	**none**	**none**
185	N-n-octanoylnornicotine	-16.26	none	none	none	none
186	N-(4-hydroxyundecanoyl)anabasine	-13.509	none	none	none	none
187	Capsaicin	-10.379	none	none	none	none
188	Harmalol	-8.933	high	none	none	none
189	Saprosmine A	-8.7616	none	none	none	none
190	Saprosmine B	-6.8486	none	none	none	none
191	13(R)-antofine-N-oxide	-4.8761	low	high	none	none
192	tylophoridicine F	-3.97	low	high	none	none
193	tylophoridicine C	-3.97	low	high	none	none
194	13(R)-14(R)-hydroxyantofine-N-oxide	-3.97	low	high	none	none
195	canthin-6-one 9-O-beta-glucopyranoside	-3.2891	low	none	none	none

196	Cephalotaxine beta-N-oxide	-3.2562	none	none	none	none
197	Cephalotaxine alpha-N-oxide	-3.2562	none	none	none	none
198	4-methoxycarbonyl-5,10-benzogquinolinequinone	-3.0999	high	none	none	none
199	Sanguinarine	-2.4707	none	none	none	none
200	Berberine	-2.2467	none	none	none	none
201	Chelerythrine	-2.1842	none	none	none	none
202	beta-carboline-1-propionic acid	-1.9151	low	none	none	none
203	Papaverine	-1.7454	none	none	none	none
204	7-methoxy-beta-carboline-1-propionic acid	-1.706	low	none	none	none
205	9-methoxycanthin-6-one 3N-oxide<NL>	-0.99092	none	none	none	none
206	Harmane	-0.96557	high	none	none	none
207	Ellipticine	-0.96557	high	none	none	none
208	Harmine	-0.92948	low	none	none	none
209	liriodenine	-0.92304	none	none	none	none
210	Cis-Zeatin	-0.51868	high	high	low	high
211	Rohitukine	-0.1543	none	none	none	none
212	Holamine	-0.066098	none	none	high	none
213	**Marcanines A**	**0.016126**	**none**	**none**	**none**	**none**
214	**Matrine**	**0.2541**	**none**	**none**	**none**	**none**
215	Brucine N-oxide	0.26167	none	low	none	none
216	canthin-6-one	0.35032	low	none	none	none
217	(R)-cryptopleurine	0.52242	low	high	none	none
218	**(-)-anonaine**	**0.61**	**none**	**none**	**none**	**none**
219	15-alpha-Hydroxyholamine	0.635	none	none	high	none

220	3-alpha-Amino-14-beta-hydroxypregnan-20-one	0.82077	none	none	high	none
221	9-methoxycanthin-6-one	1.24	high	none	none	none
222	Lycoricidinol	1.313	none	none	high	none
223	17-epi-N-Demethylholacurtine	1.5367	none	none	high	none
224	N-Demethylholacurtine	1.5367	none	none	high	none
225	**Vincamine**	**1.8025**	**none**	**none**	**none**	**none**
226	**Physostigmine**	**1.9297**	**none**	**none**	**none**	**none**
227	**9-hydroxycanthin-6-one**	**2.1077**	**none**	**none**	**none**	**none**
228	Holacurtinol	2.1445	none	none	high	none
229	Neocryptolepine	2.3562	none	none	high	none
230	**Harmol**	**2.6265**	**none**	**none**	**none**	**none**
231	**Cryptolepine**	**2.9343**	**none**	**none**	**none**	**none**
232	**Corydaline**	**3.1151**	**none**	**none**	**none**	**none**
233	7-desmethyltylophorine	3.1309	low	high	none	none
234	6-O-desmethylantofine	3.1309	low	high	none	none
235	Isocephalotaxine	3.1454	none	none	high	none
236	Tylocrebrine	3.1505	low	high	none	none
237	Isotylocrebrine	3.1505	low	high	none	none
238	Antofine	3.1505	low	high	none	none
239	(S)-tylophorine	3.1505	low	high	none	none
240	(S)-isotylocrebrine	3.1505	low	high	none	none
241	(R)-tylophorine	3.1505	low	high	none	none
242	(R)-antofine	3.1505	low	high	none	none
243	11-beta-hydroxycephalotaxine beta-N-oxide	3.1505	low	high	none	none
244	**Lycorine**	**3.1877**	**none**	**none**	**none**	**none**

245	**Rhyncophylline**	**3.3474**	**none**	**none**	**none**	**none**
246	(S)-tyloindicine I	3.3476	none	none	high	none
247	(+)-tylophorinidine	3.5386	low	high	none	none
248	6-O-desmethylsecoantofine	3.6885	none	none	high	none
249	Secoantofine	3.7051	none	none	high	none
250	Psychotrine	3.9783	none	high	none	high
251	Tubulosine	4.1737	none	high	none	high
252	tyloindicine G	4.3085	none	none	high	high
253	tyloindicine F	4.3085	none	none	high	none
254	**Hydrastine**	**4.3358**	**none**	**none**	**none**	**none**
255	Holacurtine	4.3905	none	none	high	none
256	17-epi-Holacurtine	4.3905	none	none	high	none
257	**Isostrychnine**	**4.5247**	**none**	**none**	**none**	**none**
258	**Strychnine**	**4.5843**	**none**	**none**	**none**	**none**
259	Brucine	4.6438	none	low	none	none
260	Reserpic acid	4.7169	none	none	high	none
261	**Evodiamine**	**5.1076**	**none**	**none**	**none**	**none**
262	**Camptothecin**	**5.3292**	**none**	**none**	**none**	**none**
263	9-Aminocamptothecin	5.3457	high	low	none	none
264	**Reticuline**	**5.29**	**None**	**None**	**None**	**None**

Note: Bold entries were taken for further screening

Table S3: Docking results of iGEMDOCK algorithm

S. No.	Compounds	iGEMDOCK (kcal/mol)
**Ref**	Artemisinin	-91.3722
**1**	**Axelopran**	**-125.326**
**2**	**Nicomorphine**	**-122.544**
**3**	**Fentonium**	**-121.709**
**4**	**Namitecan**	**-121.464**
**5**	**Morphine glucuronide**	**-119.945**
**6**	**Rubitecan**	**-119.292**
**7**	**Aclidinium**	**-116.089**
**8**	**K-252a**	**-115.782**
**9**	**7-ethyl-10-hydroxycamptothecin**	**-115.559**
**10**	**Tonapofylline**	**-113.324**
**11**	**Oxitropium**	**-112.543**
**12**	**Topotecan**	**-110.615**
**13**	**Hydrastine**	**-110.116**
**14**	**Tretoquinol**	**-110.106**
**15**	**Theodrenaline**	**-109.685**
**16**	Bicuculline	-109.455
**17**	**Belotecan**	**-108.553**
**18**	**10-hydroxycamptothecin**	**-108.289**
**19**	**Bamifylline**	**-107.61**
**20**	**Xanthinol**	**-107.509**
**21**	**Nalfurafine**	**-106.569**
**22**	**Naxifylline**	**-106.121**
**23**	**Variolin B**	**-104.742**
**24**	**Diflomotecan**	**-103.702**
**25**	**Ipratropium**	**-103.666**
**26**	**Lycorine**	**-102.344**
**27**	**Methscopolamine**	**-102.245**
**28**	**Hyoscyamine**	**-101.993**

**29**	**Camptothecin**	**-101.781**
**30**	**Rhyncophylline**	**-101.492**
**31**	**Noscapine**	**-101.414**
**32**	**Tiotropium**	**-101.042**
**33**	**7-Hydroxystaurosporine**	**-100.894**
**34**	**Benzoylecgonine**	**-99.9005**
**35**	**Furafylline**	**-99.4678**
**36**	**Quinine**	**-99.0127**
**37**	**18-methoxycoronaridine**	**-98.5351**
**38**	**Dyphylline**	**-98.2116**
**39**	**PCS-499**	**-97.0292**
**40**	**Vincamine**	**-96.9741**
**41**	**Buprenorphine**	**-96.7721**
**42**	**Prajmaline**	**-96.477**
**43**	**Evodiamine**	**-96.2743**
**44**	**Staurosporine**	**-96.2402**
**45**	**Atropine**	**-96.1418**
**46**	**Rolofylline**	**-95.7426**
**47**	**Reticuline**	**-95.6594**
**48**	**Anisodamine**	**-95.0347**
**49**	**Exatecan**	**-95.0082**
**50**	**8-cyclopentyl-1,3-dipropylxanthine**	**-94.2662**
**51**	**Cimetropium**	**-94.0635**
**52**	**Methylatropine**	**-94.0079**
**53**	**Samidorphan**	**-93.6762**
**54**	**Yohimbine**	**-93.6743**
**55**	**Cyprenorphine**	**-93.4031**
**56**	**Rauwolfia serpentina root**	**-93.2392**
**57**	**Methylnaltrexone**	**-93.142**
**58**	**Etybenzatropine**	**-93.0622**
**59**	**Deptropine**	**-92.8259**
**60**	**Isostrychnine**	**-92.619**

**61**	**Lucerastat**	**-91.9422**
**62**	**Diprenorphine**	**-91.803**
**63**	Ajmaline	-90.9876
64	Vinburnine	-90.284
65	Pholcodine	-90.2835
66	Benzatropine	-90.1175
67	Corydaline	-90.0204
68	Nalbuphine	-89.9547
69	5-Methoxy-N,N-diisopropyltryptamine	-89.7153
70	Scopolamine	-89.7064
71	Quinidine	-89.5134
72	Naltrexone	-88.9509
73	Apomorphine	-88.6905
74	Physostigmine	-88.5954
75	(-)-anonine	-87.93
76	Ioflupane I-123	-87.9
77	Strychnine	-87.4441
78	Naloxone	-87.2566
79	Anisotropinemethyl bromide	-87.1993
80	Oxymorphone	-85.8151
81	Cryptolepine	-85.7146
82	Nalorphine	-85.5843
83	Diamorphine	-85.4754
84	Miglustat	-85.0007
85	Miglitol	-84.7194
86	Marcanines A	-84.5263
87	9-hydroxycanthin-6-one	-83.946
88	Nalmefene	-83.6774
89	Oxtriphylline	-83.6001
90	Hydromorphone	-83.5136
91	Morphine	-83.4119
92	Harmol	-81.6226

93	Dihydromorphine	-80.9058
94	Pilocarpine	-80.8781
95	NS-2359	-79.9321
96	Galantamine	-79.591
97	Matrine	-78.9337
98	Hydrocodone	-78.6178
99	Migalastat	-78.5602
100	Codeine	-77.95
101	Oxycodone	-76.3101
102	Desomorphine	-75.1219
103	8-azaxanthine	-74.939
104	Drotebanol	-74.8465
105	Duvoglustat	-74.3026
106	Bromotheophylline	-73.0872
107	8-chlorotheophylline	-72.7254
108	Ecgonine	-72.404
109	Xanthine	-71.5821
110	Ethylmorphine	-71.4814
111	Dihydrocodeine	-71.3592
112	Sparteine	-68.1736
113	Nicotine	-65.4694
114	Pseudotropine	-57.0935
115	Tropinone	-51.787

Note: Bold entries were taken for further analysis

Table S4: Computational estimation of oral toxicity (LD50) and oral absorption availability (clogS)

S. No.	Compounds	AMES toxicity	Oral Rat Acute Toxicity (LD50) mol/kg	Oral Rat Chronic Toxicity (LOAEL) (log mg/kg_bw/day)	Hepatotoxicity
Ref.	Artemisinin	Yes	2.459	1	No
1	Exatecan	No	3.06	1.323	Yes
2	10-hydroxycamptothecin	No	2.418	1.738	Yes
3	Fentonium	No	2.883	1.411	Yes
4	7-ethyl-10-hydroxycamptothecin	No	2.444	1.551	Yes
5	Diflomotecan	No	2.551	1.324	Yes
6	Camptothecin	No	2.565	1.756	Yes
7	Morphine glucuronide	No	2.312	3.346	Yes
8	Rubitecan	Yes	2.789	1.494	Yes
9	Nicomorphine	No	3.172	2.149	Yes
10	Rauwolfia serpentina root	No	2.974	0.431	Yes
11	Belotecan	No	3.03	1.994	Yes
12	**Yohimbine**	**No**	**2.942**	**0.687**	**No**
13	Deptropine	No	2.711	0.414	Yes
14	Axelopran	No	2.278	2.09	Yes
15	**Hydrastine**	**No**	**2.893**	**2.319**	**No**
16	Lycorine	Yes	2.545	0.912	Yes
17	Evodiamine	Yes	2.542	1.006	Yes
18	**Tretoquinol**	**No**	**2.886**	**1.843**	**No**

19	Nalfurafine	No	3.285	0.278	Yes
20	**Vincamine**	**No**	**3**	**0.613**	**No**
21	**Aclidinium**	**No**	**2.301**	**1.028**	**No**
22	**Reticuline**	**No**	**2.296**	**1.542**	**No**
23	**Isostrychnine**	**No**	**3.087**	**2.097**	**No**
24	Diprenorphine	No	2.858	0.575	Yes
25	Naxifylline	Yes	2.618	0.239	Yes
26	Variolin B	No	2.355	2.099	Yes
27	**Noscapine**	**No**	**2.91**	**2.313**	**No**
28	**Rolofylline**	**No**	**2.751**	**0.411**	**No**
29	Cyprenorphine	No	2.847	0.609	Yes
30	Tonapofylline	No	2.638	1.103	Yes
31	Theodrenaline	Yes	2.184	2.271	No
32	Quinine	Yes	2.728	0.635	Yes
33	Namitecan	Yes	2.727	2.078	Yes
34	**Benzoylecgonine**	**No**	**1.787**	**2.051**	**No**
35	18-methoxycoronaridine	Yes	3.464	0.793	Yes
36	8-cyclopentyl-1,3-dipropylxanthine	Yes	2.835	0.457	Yes
37	**Methylatropine**	**No**	**2.624**	**2.624**	**No**
38	Samidorphan	No	2.143	2.317	Yes

Note: Bold entries were taken for analysis

Table S5:3D pharmacophore analysis using PharmaGist

S. No.	Compounds	Best Pairwise Alignment Scores
**1**	**Noscapine**	**6.64027**
**2**	**Reticuline**	**6.6333**
**3**	**Aclidinium**	**6.3347**
**4**	Benzoylecgonine	6.32994
**5**	Vincamine	6.329
**6**	Tretoquinol	6.32673
**7**	Yohimbine	5.433
**8**	Hydrastine	5.13019
**9**	Rolofylline	4.818
**10**	Methylatropine	4.52976
**11**	Isostrychnine	3.926

Note: Bold entries (top 3) were taken for MSD analysis

Table S6: Binding Free Energy calculations using MMPBSA

Complex	Van der Waal energy (kJ/mol)	Electrostatic energy (kJ/mol)	Polar solvation energy (kJ/mol)	Binding energy (kJ/mol)
**FP2-Artemisinin (Reference)**	-82.58 +/- 9.13	-7.53 +/- 8.79	33.10 +/- 11.41	-64.76 +/- 10.39
**FP2-Aclidinium**	-276.00 +/- 11.02	-25.11 +/- 6.27	122.14 +/- 9.33	-204.23 +/- 12.37
**FP2-Noscapine**	-145.10 +/- 14.16	-30.27 +/-12.09	85.36 +/-18.48	-104.44 +/- 13.80
**FP2-Reticuline**	-207.69 +/- 9.30	0.04 +/- 2.224	42.910 +/- 8.22	-183.37 +/- 10.88

Table S7: Detail of the top compounds with interacting residues, types of bond and bond distance

Compound	Interacting residues	Types of bond with distance
Artemisinin (Reference)	ALA 157	3.78, 4.99 (pi-alkyl)
	TRP 206	4.22 (pi-alkyl), 4.13 (pi-alkyl), 4.88 (pi-alkyl and alkyl), 5.09 (pi-alkyl), 2.39 (pi-sigma)
	TRP 210	4.67, 5.41 (pi-alkyl)
Noscapine	TRP 206	2.99 (carbon hydrogen), 4.02 (pi-pi stacked), 4.13 (pi-pi stacked)
	ALA 157	5.15 (pi-alkyl)
	SER 153	2.67 (carbon-hydrogen)
Reticuline	ALA 157	3.93, 4.96 (pi-alkyl),
	TRP 206	3.64 (pi-pi stacked)
	ASP 154	3.74 (carbon-hydrogen)
	VAL 152	5.46 (alkyl), 3.37 (carbon hydrogen)
	ASN 173	2.45 (hydrogen bond)
Aclidinium	GLN 36	2.32 (hydrogen bond)
	ASN 173	2.38, 2.54 (carbon hydrogen)
	HIS 174	2.53 (carbon hydrogen)
	GLY 40	2.27 (carbon hydrogen)
	ALA 175	4.78 (pi-alkyl)
	LEU 84	4.76 (pi-alkyl)
